# Measurement Method Options to Investigate Digital Screen Technology Use by Children and Adolescents: A Narrative Review

**DOI:** 10.3390/children11070754

**Published:** 2024-06-21

**Authors:** Amber Beynon, Danica Hendry, Charlotte Lund Rasmussen, Andrew L. Rohl, Rebecca Eynon, George Thomas, Sarah Stearne, Amity Campbell, Courtenay Harris, Juliana Zabatiero, Leon Straker

**Affiliations:** 1 ARC Centre of Excellence for the Digital Child, Australia; amber.beynon@curtin.edu.au (A.B.); danica.hendry@curtin.edu.au (D.H.); charlotte.rasmussen@curtin.edu.au (C.L.R.); andrew.rohl@curtin.edu.au (A.L.R.); george.thomas1@uq.edu.au (G.T.); sarah.stearne@curtin.edu.au (S.S.); a.campbell@curtin.edu.au (A.C.); c.harris@curtin.edu.au (C.H.); juliana.zabatiero@curtin.edu.au (J.Z.); 2 School of Allied Health, Curtin University, Perth, WA 6845, Australia; 3 Curtin Institute for Data Science and School of Electrical Engineering, Computing and Mathematical Sciences, Curtin University, Perth, WA 6845, Australia; 4 Oxford Internet Institute, University of Oxford, Oxford OX1 2JD, UK; rebecca.eynon@oii.ox.ac.uk; 5 Health and Wellbeing Centre for Research Innovation, School of Human Movement and Nutrition Sciences, The University of Queensland, Brisbane, QLD 4006, Australia

**Keywords:** children, screen use, technology, method

## Abstract

The role and potential impact of digital screen technology in the lives of children is heavily debated. Current evidence is limited by the weakness of measures typically used to characterise screen use, predominantly proxy- or self-reports with known inaccuracy and bias. However, robust and detailed evidence is needed to provide practical trustworthy guidance to families and professionals working with families. The purpose of this paper is to support researchers to select measurement method(s) that will provide robust and detailed evidence. The paper outlines the challenges in measuring contemporary screen use by children, using a child–technology interaction model to organise considerations. A range of different methods used to measure digital screen technology use in children and adolescents (i.e., questionnaires, diaries, electronically prompted sampling, direct observation, fixed room cameras, wearable/portable cameras, audio recorders, screen-device onboard logging, remote digital trace logging and proximity logging) are described along with examples of their use and constructs typically measured as well as a summary of the advantages and disadvantages of each method. A checklist and worked examples are provided to support researchers determining the best methods or combination of methods for a research project.

## 1. Introduction

The role of digital screen technology (‘screens’) in the lives of children and its potential impact has been heavily debated [1,2,3,4]. There is some evidence to support positive, helpful impacts for children using screens such as increasing learning capacities, higher productivity and enhanced competence in social interaction [5,6,7]. However, there is also some evidence to support negative, harmful impacts such as for physical, emotional and cognitive well-being and overall development [6,8,9,10,11]. Overall, there is relatively weak evidence which provides sufficient detail on the nature of use to support informed decision making by families for their children’s use of screens [12,13]. To better understand the potential positive and negative impact of screen use on children and adolescents—and thus to be able to provide practical evidence-based information to families—it is firstly crucial to have robust methods to measure screen use. This paper outlines some of the challenges faced in capturing contemporary screen use by children and the advantages and disadvantages of available options for measuring screen use by children.

### 1.1. Challenges in Measuring Children’s Complex Digital Screen Technology Engagement

**Figure 1:**
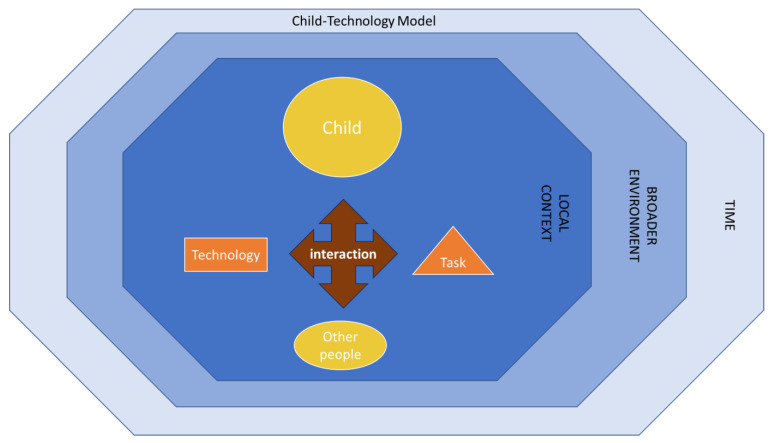
Child-Technology Interaction model.

Technological advancements are bringing growing complexities to children’s engagement with screens, creating important challenges for capturing critical aspects of contemporary screen use. Children now commonly use multiple devices and software, with differing content, for different tasks and in a variety of contexts. This contrasts with the much simpler situation when the first studies on screen use were conducted, when screen use was just television (TV) viewing of a limited number of scheduled broadcast channels. Figure 1 illustrates a conceptual model which outlines the complexity of the child-technology interactions. The conceptual model is based on human–computer interaction models [14,15] and shares aspects with earlier models [16,17], and further refined by the authors with input from community advisory groups.

#### 1.1.1. Child

In considering the **child** at the centre of the model, an important challenge is that different aspects of screen use may be important for different children. How infants and toddlers engage with technology can be vastly different to how adolescents engage with technology; therefore, different methods may be required for different age groups. Children’s gender, interests and physical, mental or social capabilities and propensities may also be important considerations. Further, different considerations, including ethical ones, may be required for children with disabilities, and for children and families from culturally and linguistically diverse backgrounds [18].

#### 1.1.2. Technology

A further measurement challenge is that the **technology** children interact with includes different hardware, software and content. A range of different technology devices with different capabilities and contents are being used by children and adolescents. Each household generally has multiple devices that can be shared. For example, typical homes in the USA have five internet-connected devices (e.g., computer, smartphone, tablet, television, etc.) [19], and young children often use other people’s devices, such as from their parents or older siblings. Many prior studies have used aggregated groups of screen use, such as combining TV viewing and mobile touch screen device (MTSD) use together, despite known differences in the potential interaction [20]. These multiple devices can also be used simultaneously with children multitasking. Adding to this challenge is that some hardware can operate multiple software programs (apps) which in turn can support multiple contents. For example, a tablet computer can operate one app for internet searching and another for video playback and both apps can present many types of content. Content on the video app, for example, may range from home-made videos to professionally produced movies. Measuring content has been shown to be important, for example pro-social or anti-social content can have different impacts [21].

#### 1.1.3. Tasks

A prominent measurement challenge is dealing with the variety of **tasks**, or purposes, for which children use screens. Technology may be used for relaxation (which is important for health), for activities of daily living such as navigation and encouraging teeth brushing, or it can be used for communication such as video chat and social media interaction. Prior studies have often separated the purpose of use into educational versus recreational [22], however, whether use is educational may not be clear cut, especially for young children. For example, a young child may play a numbers game on an app and see it as recreation whereas their parent sees it as education. Similarly, an adolescent may create music on an app for recreation but be learning about music concepts. This challenge is further illustrated by the same hardware, software and content potentially having a different purpose based on the perspective of the user or the observer. For example, what is seen as recreational by a child may be perceived as educational by their parent.

#### 1.1.4. Interaction

The complexity of the challenge for measuring children’s technology is not only dealing with the child, technology and task variety, but also the different aspects of the **interaction** of these elements within the local and broader system contexts. For example, it may be important to measure the information flow between child and technology or the physical posture assumed. The short-term aspects of interaction may also have longer term consequences that also need to be considered such as cognitive development and musculoskeletal development. Most studies have focused on the cumulative duration of interaction, with screen time forming the basis of most health guidelines [23,24,25,26]. But the effect size of screen time is increasingly being questioned [13], and other aspects of child-technology interactions are increasingly being recognised as influential [7,27].

#### 1.1.5. Other People

Children in particular are often interacting with technology together with **other people**, such as peers, siblings and parents. While this often involves people being physically in the same location, virtual co-use is also common, for example, video chat with remote family or friends [28]. Capturing significant aspects of these interpersonal dimensions is an important measurement challenge as there is evidence these dimensions influence the likely impacts of child-technology interactions. For example, co-viewing has been linked to positive psychosocial health and developmental outcomes [29,30].

#### 1.1.6. Local Context

The physical and social **local context** of child-technology interaction is diverse and a challenge for measurement. Children are interacting with technology in multiple physical contexts, such as the home, educational settings and in the community, as well as in virtual worlds. Understanding screen use in these contexts is likely to be important for observational studies of children’s natural engagement with technology. Measuring across multiple contexts can be challenging, for example parents cannot realistically be expected to detail their child’s technology use when at school. The social context is likely to be important to consider. Family practices, values and rules on access and use of screens also form an important part of the local context [31]. Measurement within laboratory studies may be less challenging given the single controlled context of use.

#### 1.1.7. Broader Environment

Studies, and therefore measurement, also face the challenge of considering the **broader environment** of children’s technology engagement, including the socio-economic, cultural and physical environment. Community attitudes, cultural practices and weather may all influence children’s interaction with technology and may need to be considered, and thus measured.

#### 1.1.8. Time

A final challenge for measuring children’s interaction with technology is dealing with the **time** of use. Engagement with technology may have different impacts depending on the time of use, for example watching an exciting program may have no impact on a child’s sleep if viewed in the morning but may disrupt sleep if viewed in the evening just before bedtime. Similarly, the impact of screen use on attention and learning in school may only relate to school day screen use and not weekend screen use. Lastly, patterns of screen use may vary across the year with school holidays, summer weather etc.

In attempting to measure screen use, researchers have most commonly used self- or proxy-reported methods such as questionnaires or diaries, that try to capture multiple aspects of the child-technology interaction [32,33]. These subjective methods of measuring screen use are generally easy to administer with low cost. However, they are subject to recall inaccuracy and reporting bias leading to overall imprecision [32,34,35,36] meaning studies based on these methods may miss and/or mistakenly claim important effects. Therefore, a major challenge for the field is to find unbiased and more precise measurement methods that can deal with the complex system within which children interact with screen based technology.

### 1.2. Study Aim

The aim of this paper is to help researchers by describing currently available methods to measure screen use by children and adolescents, and by providing guidance around determining a suitable method or combination of methods to support a particular research project.

## 2. Narrative Review Approach

A narrative review approach was used for this study as the best way to address the aim of providing practical guidance to researchers in selecting measures of screen use by children and thus enable stronger evidence from future studies. Narrative reviews provide a flexible approach to interpreting existing knowledge to bring together implications for research, and are especially suited to complex issues [37,38].

The author team conducted initial searches for measurement methods to investigate children’s screen use in a range of databases (PsycINFO (Ovid), PubMed, Wed of Science (Core Collection), CINAHL, SPORTDiscus, Embase (Ovid), MEDLINE (Ovid), Scopus and iEEE), limited to 2010 onwards with a focus on young children, which located 30,312 articles after duplicates were removed. However, this comprehensive search yielded a low number of relevant methods and failed to identify known methods that had been used to measure screen use. Four recent reviews on this area were also located. Two of these reviews found a very low number of studies that had used objective methods to measure screen use in children [32,33]. The scoping review by Browne et al. considered measurement methods for digital media use in children and adolescents and found that the vast majority of methods were proxy-reported (92%), and suggested that the greatest advances in measuring screen use will revolve around using automated data collection from devices or other software solutions [32]. The systematic review by Byrne et al. summarised the measurement methods used to assess screen time in young children (0–6 years) and found that the majority of methods were proxy-reported (completed by parents) (76.3%) via questionnaire (92.4%). None of the located articles (622 articles) within the systematic review used a device-based method to measure screen time [33]. Byrne, et al. [33] highlighted the challenge of locating studies that measured just the simple construct of screen time, as information on measurements of screen time was often missing from the titles and abstracts, particularly if screen time was not a primary outcome measure. The systematic review by Perez et al. [39] focused on measures of screen media use for participants of any age, that had been validated by direct observation or video observation. They noted poor validity for proxy- or self-reported measures and extra difficulties in using technological measures of screen use by young children. The extensive narrative review by Barr et al. [40] covered many aspects of child development and digital media use, including proposing a toolkit comprising questionnaire, time use diary, passive mobile device sensing App and electronically prompted sampling.

Therefore, the range of currently available methods that could be used to measure digital screen technology use in children and adolescents was identified by the authors from their database searches, the recent published reviews and review reference lists, as well as methods known to the authors based on their diverse research fields across science, engineering, humanities and health. The current study also presents a discussion of the advantages and disadvantages of each method determined by the authors, to provide guidance for researchers to determine the best method or combination of methods to support a research project.

Whilst valuable evidence can be obtained from qualitative methods such as by using semi-structured interviews to capture reasons for overuse of digital tools by young children [41], to explore adolescents’ perceptions on their patterns and influences of mobile device use [42] or to examine parents’ views of their child’s screen-viewing time [43], this review focuses on quantitative methods.

## 3. Summary of Different Measurement Method Options

This narrative review covers the following measurement method options: self-/proxy-reporting, direct observation, recording devices, onboard logging and screen recording, network traffic logging of digital data traces, proximity logging and other specialised devices. The range of different method options included was based on iterative and purposive searches of the literature and on expertise of the review team representing a range of science, health and humanity disciplines. This review focuses on methods that can be used for capturing naturalistic child-technology interactions (e.g., use in the home), but the methods may also be useful for other contexts (e.g., laboratory studies). To provide a practical understanding for researchers, some key features of each method are described along with some examples of when the method has been used previously (focusing on children and adolescents), and typical constructs collected with the method. The potential advantages and disadvantages of each type of method are also summarised in Table 1.

### 3.1. Self-/Proxy-Reporting

The vast majority of observational studies on child-technology interaction to date have used self- or proxy- report methods such as questionnaires or diaries.

#### 3.1.1. Questionnaires

Questionnaires typically collect retrospective recall of screen use over a specific period in either paper or electronic formats. Questionnaire items can include open ended or close ended questions. Online questionnaires can have a wide reach as they allow for collection of data within a fairly short period of time from a diverse and substantial number of people without geographical barriers [44], and therefore can potentially include a wide range of participants and representative samples. Psychometric data (evidence for reliability and validity) are available for some, but not all [34,45]. Some studies have reported reasonable validity for self-/proxy- reporting, for example a study of 9- to 10-year-old participants found end-of-day reports of their exposure to information and communication technology were comparable with data from real time direct observations [46]. In contrast, another study comparing parent reported duration of child’s device use to data logged on mobile devices found that only 30% of parents were considered accurate reporters [47]. Different aspects of child-technology interaction may be more accurately reported. For example parent reports of the content of media use (e.g., child’s favourite apps or TV shows) may be more accurate that reports of duration of use [34,35]. A recent systematic review and meta-analysis of discrepancies between self-reported digital media use and device logged use highlighted the concerns about the validity of self-reported findings as they were rarely accurate [48]. As noted earlier, of particular concern is not just the unbiased recall inaccuracy but the potential for bias due to social desirability.

Examples of questionnaires used to measure screen use include Technology Use Questionnaire (TechU-Q) [49], TV viewing as part of the Youth Risk Behavior Survey Questionnaire [50,51] and Child Sedentary Activity Questionnaire [52]. Many studies have used just a single item for duration of screen use (e.g., [11]. A large number of questionnaires conflate exposure and outcome by assessing ‘problematic’ screen use, for example, Addiction Profile Index: Internet Addiction Form [53], Behavioural Addiction Measure Video Gaming [54], Bergen Social Media Addiction Scale [55], Game Addiction Scale [56] and Problematic Internet Use Questionnaire [57]. Typical constructs collected cover child, technology, task, other people and include duration of screen use, which devices are owned by participants, co-viewing and whether use is problematic.

#### 3.1.2. Diaries

Diaries collect time use data over a period of time, and like questionnaires may be in either paper or electronic format. Participants are typically provided with a graphical representation of the day, or part thereof, and for each time period (sometimes 5 or 15 min blocks) report aspects such as the activity being performed and the location of that activity. Recall periods may be shorter for diaries than for questionnaires and thus diaries may be more accurate [58]. In a large scale study, many participants provided 2 days of data, but longer recording was deemed too great a burden [59]. Some evidence for accuracy has been reported by comparing sedentary task time reported in diaries to that measured by sensors [60].

Examples of diaries used to measure screen use in children include Light Time-Use Diary [59], Multimedia Activity Recall for Children and Adults (MARCA) [60] and as part of a combined methods approach called Comprehensive Assessment of Family Media Exposure (CAFÉ) [61]. The Light Time-Use Diary has been used as parent-reporting on preschool children in the Longitudinal Study of Australian Children to collect screen use and other activities and the location of that activity [59]. MARCA was used to collect self-reports of different types of screen use, such as TV viewing and playing electronic games at a video game centre, by 643 14-year-olds for a minimum of 7 days [60]. The CAFÉ time use diary was parent completed for 24 h diary in 15 min blocks to capture content and context of media use [61].Typical constructs collected using dairies cover child, technology, task, other people, time and include duration of screen use, devices used and time pattern of use.

#### 3.1.3. Electronically Prompted Sampling

Electronically prompted sampling methods, sometimes called Ecological Momentary Assessments, are technologically aided diary systems in which participants are prompted either at a random time or at a set time of day by text message or app notification [62,63,64,65]. The prompt typically asks the participant to report what they are doing, or feeling, at that precise time. A systematic review found that electronically prompted sampling can be successfully used with children from approximately 7 years of age; however, adaptions may be necessary for younger children [65].

Examples of electronically prompted sampling include the following: to capture screen use by children and adolescents including TV viewing and mood when watching TV among a sample of adults and children ≥ 10 years old [62,63,64], and to assess current activities (e.g., watching TV/movies, playing video games and physical activities) in a sample of 121 9- to 13-year-old children [66], and associations between mood and social media use in 55 adolescents [67]. Typical constructs collected cover child, technology, task, time and include duration of screen use, devices used and time of day of use.

### 3.2. Direct Observation

Researcher direct observations of child-technology interaction over a period of time in the participants’ settings is often used as the reference standard for measuring screen use. Observation can be in-situ in real time or later viewing of video recordings (see next section). Therefore, there is a level of intrusiveness that can create participant discomfort despite researchers being respectful of privacy [46]. There may be reduced ecological validity because the presence of the observer may influence the child’s behaviour [58,68,69]. Reactivity may be reduced if the objective of measuring screen use is concealed. For example, Krugman et al. [70] observed screen use by students in their homes under the guise of examining homework, with the true purpose revealed on study completion. Although observed children from varying socioeconomic background could be captured, the high researcher burden means large (and therefore generalisable) samples are unlikely [69]. Similarly, although observations could occur in different environments and different times/seasons, the high researcher burden and intrusiveness can impede acquisition of repeated measures. For example, previous research has suggested that at least 6 to 15 days are required to acquire reliable results of habitual television viewing or physical activity [71,72].

Examples of direct observation studies of children’s screen use include the following: observing minutes of screen time in children with a mean age of 7.8 (SD: 1.8) years during an after school program [73], observing TV viewing by 3- to 4-year-olds at home for 6–12 h/day for 2.5 days [71] and observing activity patterns (including TV viewing) in 4-year-olds at home and school (recess) [74]. Typical constructs collected cover child, technology, task, other people, local context and include duration of screen use, devices used, task of screen use and contextual information such as co-viewing [69].

### 3.3. Recording Devices

Audio-visual recording is an important group of methods available for researchers and includes fixed room cameras, wearable or portable cameras, and audio devices. Cameras and audio devices have the ability to capture a variety of screen devices, sometimes with minimal burden to participants [58]. Coding recordings may be less burden than in-situ direct observation and thus longer observation periods may be possible. Recordings can also be played back to participants to gain reflections about screen use from participants. A clear advantage of mobile recording devices is the ability to capture various locations; however, recording is not allowed in some places such as banks, airports and public swimming pools. Analysis of the data including coding of the images and sound from devices can be time consuming and therefore a high researcher burden [58,69]. Further, wearing a device or taking recordings with a portable device can be seen as burdensome by participants. The use of recording devices also brings ethical concerns including participant privacy and third party consent [75]. Images and sounds may be recorded which the child/family may not want others to see or hear. Studies have reduced concerns about participant privacy by allowing participants to stop recording at certain times (by taking off the device or turning it off) or deleting some recordings at the end of data collection. Recording of people not involved in a study and who have therefore not consented needs careful consideration, including safety concerns if non-participants accost the participant [76]. Automated blurring of non-participant faces has been used to alleviate this concern. Mobile devices can also create other problems including comfort and security of attaching the device to the participant, participant concerns for damaging the device or themselves, movement blurring of images or noise, and limited battery life [76,77].

#### 3.3.1. Fixed Room Cameras

A camera fixed in one location can capture screen use within that local context, for example with multiple cameras used to capture different rooms in a home. Cameras are often set up to record continuously [78,79] although they could be triggered by a person moving into view or when technology is turned on [80]. Information is limited to activities that appear within the field of view of the camera, which is a limitation given the increased portability of devices as they may be moved out of view [69]. Depending on the view of the camera and the image clarity and resolution it may or may not be possible to capture screen content, facial expression, along with local context information such as co-viewing [81].

Example studies using fixed room cameras include the following: a pioneering study by Allen [82] installed time-lapsed cameras in 95 families’ homes that recorded the TV screen at 4 frames/min and found differences between the recorded results and the self-reported results in dairies; a home-based time-lapse video camera has also been used to record TV viewing for a 10 day period in 5-year-olds [80], and 9-year-olds [81]. More recently, algorithms based on video were developed for facial recognition and gaze angle determination in a proof of concept for measuring children watching television [83]. Typical constructs collected cover child, technology, task, other people, local context, time and include duration of screen use within a set location, TV device use and co-viewing.

#### 3.3.2. Wearable or Portable Cameras

Wearable cameras can be mounted on a chest harness, head band or suspended on a lanyard around the participant’s neck, with different movement and comfort issues related to each method of attachment. In comparison to fixed room cameras, wearable cameras usually capture what the child is looking at, but like fixed room cameras can record time-lapsed still images or videos. Portable cameras are handheld recording devices where typically a parent or caregiver records a view of the child and their technology interaction or other activity [84]. This creates a greater burden to parents or caregivers but also enables their control over what to capture, alleviating some privacy and third-party concerns. Wearable and portable cameras are also at risk of hardware damage when used in daily life situations. Wearable and portable cameras can provide location context information [58] across multiple contexts in a child’s life.

Examples of wearable camera studies include the following: SenseCam wearable cameras worn during waking hours for 3–5 days taking 3–10 images per minute to capture screen use in a sample of adolescents and adults [85] and Autographer cameras worn around the neck on a lanyard recording every 15 s for 2 days to capture screen-based activities (as well as dietary behaviours and physical activity behaviours) in a sample of 14 children (9–11 years) [76]. Thomas et al. used wearable cameras in a study of 10 adolescents (mean age 15.4 years) with adolescents wearing the device on 3 school evenings and 1 weekend day with images taken every 10 s to capture screen use type and context [86]. Typical constructs collected cover child, technology, task, other people, time and include duration of screen use, devices used (but has mainly been used for TV viewing duration) and some contextual information about screen use.

#### 3.3.3. Audio Recorders

Wearable or fixed room digital audio recording devices have been used to capture sound from TVs and radios, as well as study participant talk. A fixed room audio device may struggle to capture the required data (depending on the environment and distance from the participant) as clearly as a wearable device, although wearable devices may record movement artefact noise. Recordings can be analysed by researchers through direct listening and coding, through transcription for text analysis or more sophisticated automated analysis including speech recognition software. Software has been used to identify sound from screen technology [87], with recent advances in artificial intelligence and transcription achieving higher accuracy to discriminate between participant and screen speech [88] compared to earlier methods [89]. However, software is generally unable to distinguish between types of technological input (i.e., TV versus radio) [90], or to discriminate when the TV noise is foreground or background [89]. Audio devices obviously provide no information on screen interactions which are not audible.

Examples of audio recordings that have been used previously to capture children’s screen use include the following: using the Language ENvirnoment Analysis (LENA) system to capture conversations and electronic media exposure in children 12–36 months of age [90], capturing exposure to electronic noise for 1 day every 6 months in a sample of children 6 to 24 months of age [91] and measuring audible television in a sample of 2- to 48-month-old children [89]. Typical constructs collected cover child, technology, task, other people and include duration of screen use and conversations about screen exposure.

### 3.4. Screen-Device Onboard Logging

Screen devices themselves can provide methods for measuring their use including onboard automatic logging of internet traffic or app use or onboard manual logging. Data traffic logging (either directly or through battery use as a surrogate measure) and screen recording (both video and still image). Measurement apps based on automatic logging use (either from the smartphone/tablet manufacturer or independent software company) can measure device use: duration, frequency, time, general app type and app status (foreground, background etc.) [61] including short bursts of exposure of mobile phone use [61], and which web pages are being visited and for how long [92]. Device operating software can automatically log battery use as a surrogate measure of device use. Manual screenshots taken by the participant/parent can provide information on battery use, app use and on-screen actions and be submitted to the research team via online survey. Screen video recordings can also provide information about on-screen actions. Data per device may be more difficult to link to a particular participant if the device has more than one user [19] as current apps typically cannot identify the user. For example, a study of mobile touch screen device use by young children (0–3 years) reported 61–70% of devices had been shared [47]. As many young children do not have their own device and just tend to share devices, this method could be unsuitable for younger children [47]. There are currently two main mobile touch screen device operating systems (iOS and Android) which have different capabilities to log data, meaning researchers may not be able to acquire the same data from a broad spectrum of participants. Onboard device logging also does not capture all types of screen use (e.g., television, game consoles) [47]; therefore, it is unable to capture the full scope of screen exposure [19].

Examples of device-based logging and recordings studies include the following: onboard device logging to capture children and adolescents’ screen use in a study where mobile device sampling on 3- to 5-year-olds captured phone use with an app Chronical for Android phones and battery screenshots for iPhones [47]. In other studies, smartphone (Android) use among 18- to 33-year-olds was captured using a researcher developed app ‘Fun in a Box’ [93], and a smartphone use tracking app (Effortless Assessment of Risk Stats (EARS)) was used over 4 weeks on a sample of 67 participants 11–12 years old [94], and battery use of smartphones was captured in a sample of adolescents 12–15 years old [95]. A further example used the XMobiSense app to capture the number and duration of voice calls, text messages and amount of data transfer in mobile phone use by 466 participants 10–24 years old (mean age 18.6 years) [96]. In an example of rich detailed data collection, Ram et al. [36] captured smartphone screen shots from 4 adolescents every 5 s the smartphone was activated over several months (some 500,000 images). They then used a combination of human coding and machine learning to examine the applications viewed, consumption versus production interaction, food-related content and emotion/sentiment. Typical constructs collected cover child, technology, task, time and include capturing information about which apps and webpages were being used and for how long on certain devices as well as number and duration of voice calls and messages.

### 3.5. Remote Digital Trace Logging

As with screen device onboard logging, measuring screen use can also be done at the home router, internet service provider or digital platform (e.g., social media) levels by collecting digital trace data, that is, data that are generated as people interact with any kind of digital platform via a networked device. Interaction here includes all the ways that people may actively engage with a digital platform, whether this is through, for example, typing or drawing to create text or a picture, talking to a voice assistant, recording and uploading audio, photos, video and/or enabling geo-location. As such, this includes data related to the interaction actions (e.g., clicks) and the content of that interaction (such as text or picture) [97]. It is often referred to as ‘Big Data’ or digital footprint data, and is typically not only huge in volume but also high in velocity, being created in or near real-time, and exhaustive in scope [98]. Depending on the approach used, data trace logging can capture use across all internet-connected devices in the household. Data can be collected on an individual, a small cohort or on a huge population. The duration and frequency of the type of activity such as phone calls, messages and websites visited can be collected, along with the pattern of internet interactions, such as who is contacted, which types of websites are visited, which social media groups and individuals are visited and what is ‘liked’ on social media and what comments are made. However, there are challenges in capturing passively viewed content (i.e., where no action is taken on the page such as when someone is reading text or simply doing something else) [12]. Further, some internet interactions are end-to-end encrypted requiring a key to decode the content of the interactions; thus, it is not always possible to capture all of the relevant information, though even the meta-data about the amount of internet traffic and time of interactions may be useful. As with onboard device methods, the internet use may be difficult to link with an individual user, particularly when working at scale. Privacy issues are central, as with other measurement methods, and the use of this type of data without specific participant consent is of current community concern. Indeed, there are notable differences between commercial and academic practices in the collection and use of such data, with varied perspectives on what constitutes ethical practice.

Examples of internet digital trace logging include the following: a study on adults using server log data of outgoing voice calls and SMS that found participants generally overreported when self-reporting daily usage compared to log data [99], and identifying aspects of an educational game that best related to enhanced learning outcomes [100]. Typical constructs collected cover child, technology, task, time and include capturing information including number and durations of voice calls and frequency of text messages and specific aspects of interaction with an app.

### 3.6. Proximity Logging

Radio-frequency identification (RFID) can be used to detect when a participant (wearing a chip) is near to a screen device (also with a chip attached). The chips can be small (fingernail size) and thin (paper thick) so can be attached as a sticker. Chips are also cheap and regularly used in community participation running and cycling events to clock start and finish times for participants. Information is only available when the participant is in close proximity to the screen, or to other participants if each family member wears a chip. The method is therefore unable to measure whether the screen is on, nor whether the user is interacting with the screen, nor is it able to capture the content of screen use. An example of proximity logging has been to capture TV viewing during 2 consecutive days, in a sample of 7 children with mean age 10.7 years (SD: 2.1) [101]. Typical constructs collected cover child, technology and other people and include capturing information including specific device (such as television) ‘use’ duration, and co-viewing.

### 3.7. Other Systems

There are a number of other systems that have been used in the past to monitor screen use and/or restrict screen use. These systems included special hardware and/or software and provide ideas for what measurement methods could offer.

The Nielson People Meter monitored what TV program was being viewed and who was watching. As a participant started watching TV, a light flashed on the meter controller reminding them to press their assigned button (to log in). When the participant had finished watching, the participant pressed the button again to log out [102]. As this required participants to log in and out this is not always done correctly [103]. Participant fatigue has been observed with the recorded viewing time of participants reducing over the days of the study [104]. However, short term monitoring has had high levels of compliance [103]. The Nielson People Meter has mainly been used previously for TV broadcasting analysis for children as young as 2 years of age to capture content and co-viewing [102]. The data regularly collected survey with representative sampling has been commercially available, but was expensive. The system was developed when the screen environment was much simpler and focused on broadcast/cable TV, which does not represent contemporary screen use by children and adolescents.

The Arbitron Portable People Meter captured similar information but was based on a small device worn by the participant which accessed an inaudible code embedded in the audio stream of audio and video programming. This system has been used to measure advertising exposure including for participants from 12 years of age [105]. As with the Nielson system it did not capture the breadth of screen use by contemporary children.

TV Allowance was a semi-automated device that monitored TV and computer monitor use, through a power cord. In order to turn on the device, the participant entered an individual four-digit code. The cumulative use of each device was then calculated and power withheld from the devices if the individual had already consumed their time allowance. TV Allowance has been used to capture screen use by children in home-based studies including TV viewing in samples of 4- to 7-year-old children [106] and 3- to 5-year-old children [107]. Typical constructs collected include TV and computer duration of use and it can capture co-viewing if each family member enters their code when they start and end watching/use.

Other methods that may be useful in future studies in children include software systems for tracking computer keystroke and mouse activity across a whole office workplace that were developed in the wake of a risk in upper limb musculoskeletal disorders in the 1980s [108], and eye tracking to identify what components of a computer screen were attracting user attention [109].

**Table 1:** Method options to investigate digital screen technology use by children and adolescents.

Types of Measure and Example Studies	Methods	Advantages	Disadvantages
**Self-/Proxy-(e.g., Parent, Teacher etc.) Reporting**			
*Questionnaire* Howie et al., 2020 [49] He et al., 2009 [52] Kwon et al., 2024 [11]	Retrospective recall of screen use through paper or electronic format.	Low burden to participants (if short) Low cost Large scale possible Wide reach possible Can capture range of constructs including use, interaction and context Data quickly ready for analysis (if electronic)	Subject to recall inaccuracy and reporting/social desirability bias leading to overall imprecision Online only format may bias sample Only proxy-report for young children
*Diary* Tey et al., 2007 [59] Straker et al., 2013 [60] Barr et al., 2020 [61]	Recall of screen use across day through paper or electronic format.	Reduced inaccuracies with shorter recall and prompts provided by structure of day compared with questionnaire Relatively low cost Large scale possible Wide reach possible Can capture range of constructs including use, interaction and context	Subject to recall inaccuracy and reporting/social desirability bias leading to overall imprecision Higher degree of participant burden compared with questionnaire Data processing more complicated than questionnaire Only proxy-report for young children
*Electronically prompted sampling* Larson et al., 1989 [64] Dunton et al., 2011 [66] Nareim et al., 2022 [67]	Instant recall of screen use or associated factors in response to Text or App messages to participant.	Instant recall improves accuracy Large scale possible Wide reach possible Can capture range of constructs including use, interaction and context	Subject to reporting/social desirability bias leading to overall imprecision Higher degree of participant burden compared to questionnaire More intrusive than questionnaire and diary Prompting system may be costly Requires participant to have a text or message receiving device (e.g., smart phone and mobile signal) Data processing more complicated than questionnaire Only proxy-report for young children
**Direct observation** Lee et al., 2014 [73] DuRant et al., 1994 [71] McKenzie et al., 1992 [74]	Conemporaneous observation and recording screen use by Trained observer in their natural environment through paper or electronic format.	Contemporaneous recording improves accuracy Less potential for reporting bias from ‘independent’ observer Can capture rich detail for a range of constructs including use, interaction and context Can be used for children of any age	Intrusive to child and family Presence of the observer may influence child and family’s behaviour High researcher data collection burden so impractical for large scale and wide reach Data processing can be complicated
**Recording devices**			
*Fixed room cameras* Anderson et al., 1985 [80] Borzekowski 1999 [81] Vadathya et al., 2022 [83]	Contemporaneous fixed camera recording still images or video capturing screen use within one setting per camera.	High accuracy and low bias Low burden to participants May be less intrusive than direct observation Less burden on researchers for data collection than direct observation Less burden on researchers for human coding than direct observation as can fast forward recordings Can capture rich detail (similar to direct observation) for a range of constructs including use, interaction and context Can be used for children of any age Potential lower analysis burden using machine learning	Intrusive to child and family Third-party privacy issues. Likely limited to small scale Limited to activities within the range of view of fixed camera High researcher burden for human coding Data processing can be complicated Large data sets may create management and file use issues
*Wearable or portable camera* Kerr et al., 2013 [85] Everson et al., 2019 [76] Thomas et al., 2022 [86]	Contemporaneous wearable camera (attached to participant usually on chest or head or on neck lanyard) recording still images or video in the field of view of the participant. Contemporaneous portable camera (typically handheld by parent or researcher) recording still images or video.	High accuracy and low bias Can capture a variety of digital technology devices Wearable camera can capture what the child sees and can provide some setting information Portable camera can capture more context information Can capture across multiple settings Can be used with children across a wide age range Potential lower analysis burden using machine learning	Intrusive to child and family Third-party privacy issues Moderate child burden of wearing camera Moderate parent/ researcher burden for using portable camera Likely limited to small scale If worn on chest the camera field of view may miss important information, and be uncomfortable for older female children If worn on head may create discomfort to the participant Limited information within the view of camera. Battery life of cameras can be short Can not be used in some locations High researcher burden for human coding Data processing can be complicated Large data sets may create management and use issues
*Audio recording* Ambrose et al., 2014 [90] Brushe et al., 2023 [91] Christakis et al., 2009 [89]	Contemporaneous fixed room or wearable device capturing sound (screen technology as well as voices of participants and other people nearby).	High accuracy and low bias May be more acceptable to families than cameras Typically, a longer battery life than cameras (depending on the device) Can capture all electronic sound and participant and other voices that may be occurring concurrently in the vacinity Can be used with children of any age Potential lower analysis burden using machine learning	Invasive to child and family privacy Third party privacy issues Not able to capture screen use that is not audible Software may have limited ability to distinguish between devices and whether device noise is from screen child is engaging with or just in the background Battery life may limit data collection Likely limited to small scale Data processing can be complicated Some devices require cloud software for analysis which raises ethical concerns
**Screen-device onboard logging** Radesky et al., 2020 [47] Goedhart et al., 2018 [96] Ram et al., 2020 [36]	Contemporaneous manual or automated onboard capture of smart phone or tablet use with app or screen recording.	Automated logging has high accuracy (if sole user of device) and low bias Could be used at large scale and broad reach Able to capture short bursts of exposure e.g., of mobile phone use Automated logging can measure duration, frequency, time, general app type and app status (foreground, background etc) Manual screen recording may not require loading specific app Potential lower analysis burden using machine learning	Invasive to personal privacy Manual screen recordings may be biased and lack timestamp data Manual screen recording creates participant burden, and researcher burden Currently not able capture all types of screen use (e.g., television, game consoles) May not identify user of device Requires loading app onto participant’s device Automated logging apps may only work on some devices Data processing can be complicated Large data sets may create management and use issues
**Remote digital trace logging** Boase et al., 2013 [99] Lui et al., 2023 [100]	Contemporaneous automatic capture of network traffic at router, internet service provider or platform.	High accuracy and low bias Low burden to participants Can be low cost Can capture at very large scale and with very wide reach Can capture rich detail of interactions Can potentially be used with children of any age depending on legal and cultural contexts Potential lower analysis burden using machine learning	Invasive to personal privacy Can only capture certain types of data May not identify user of device May require agreement of network/platform Data processing can be complicated Large data sets create management and use issues
**Proximity logging**	Contemporaneous detection when a participant is near to a screen (when both have chips attached) using radio frequency identification.	High accuracy and low bias Low burden to participants No identifying data collected Can capture proximity to range of devices Can be used for multiple devices and people within one context Can be used with children of any age	Only records proximity, not actual use

## 4. Potential Future Methods

As screen technology develops so do the potential technical advances in measurement. These advances may be able to reduce research and participant burden, and more accurately capture child and adolescent screen use.

In future work, there is significant potential for exploring the possibilities of digital trace data [12]. However, there are several challenges that need to be addressed. Current work tends to focus on the activities on one device (e.g., tracking mobile phone use via screen capture and tracking software) or tends to focus on one digital platform (e.g., via data collection via an application programming interface (API), data scraping or direct collaboration with owners of digital platforms to use the digital trace data they have already or are collecting). It is rarely representative (c.f. [110,111]). It can sometimes be a relatively high burden for participants (e.g., data ‘donation’ where participants request their information from companies and then share this information directly with researchers). There are other problems too, such as issues related to validity, reliability and bias; data privacy, informed consent and the legalities of working with such data for those under 18; and questions about the environmental cost of using data intensive technologies (e.g., [112,113]) that need to be considered.

Nevertheless, given the importance of understanding screen use in greater depth, such an endeavour is worth further exploration and debate. Technically, some of the issues could be addressed in future work, through for example, accessing people’s accounts on social media and other digital platforms with their permission via APIs which allow the participant to give ‘read only access’ to their account to researchers, which has the advantage that behaviours are captured across multiple devices. Linking such forms of digital trace data collection to other methods, such as representative surveys (to capture important socio-demographic or attitudinal variables) or government data (e.g., on educational achievement or health) could also be valuable to better infer the social, educational and health implications of screen use. Closer collaborations with technology companies, to enable the design and collection of digital trace data that is academically important (including, for example, pop up surveys in games to better understand motivations for game play) could also be fruitful, within a wider data collection strategy that triangulates data from varied sources.

As data collection opportunities become ever larger and more complex in scope, there are also questions of how best to analyse the data collected [12,36]. Machine learning software could be used to combat the high researcher burden for the analysis of screen use via images, movies or audio. For example, the YOLO (You Only Look Once) family of computer vision models have been rapidly evolving since version 1 was introduced in 2016 [114]. Version 8, released in 2023, is able to detect, segment, track and classify objects in better than real-time in an image or movie. Furthermore, YOLO is able to estimate pose, potentially allowing for changes in posture whilst using digital devices to be monitored. Similar advances are being made in audio classification and transcription.

There may also be technical approaches to addressing some of the concerns around privacy and data protection. For example, although there is great potential to use cameras to record children’s use of digital devices, there are a number of significant privacy issues associated with using cameras. Light Detection and Ranging (LiDAR) technology provides a point-cloud based 3D map of the area being monitored. Thus, LiDAR could be used [115] to determine screen use or activity density within a set location without capturing identifiable information therefore reducing the ethical considerations of privacy particularly within public locations.

Despite the potential, the opportunities for researchers to collect rich digital trace data are steadily being eroded, as technology companies protect their commercial interests. Politically, academic researchers need to become far more powerful actors in shaping the direction of how digital trace data are collected, analysed and used. Further, given the nature of digital trace data, decisions about when and how to collect such data need to be made in collaboration with the public, to ensure the needs, interests and perspectives of all stakeholders are designed into any approach. We would therefore suggest co-design approaches (e.g., [116]), working with families, legal experts, childhood educators and others when developing future approaches in this domain.

Future research on developing better methods for assessing screen use by children and adolescents could therefore include developing validated low participant and researcher burden measures that address the relevant aspects of child-technology interaction.

## 5. Researcher Checklist for Measurement Method Selection

**Figure 2:**
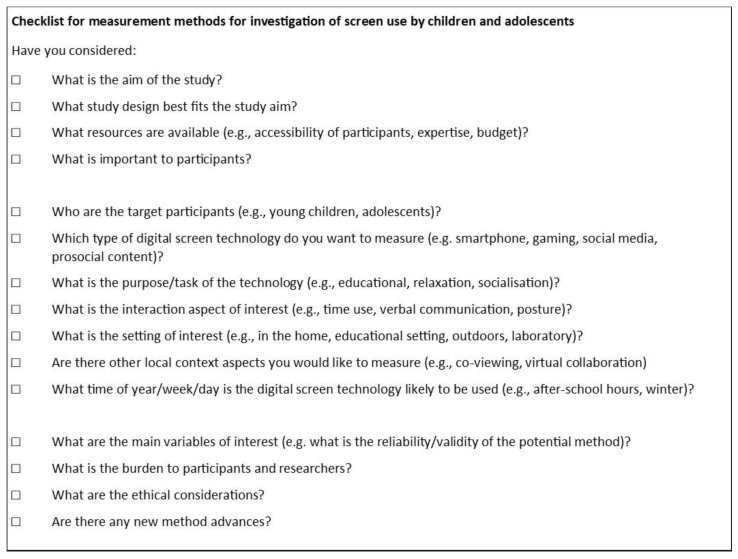
Researcher considerations checklist.

When designing a research project to investigate associations between screen use and child development there are many considerations that researchers should contemplate. The child-technology interaction model present in Figure 1 can serve to remind researchers of the different aspects which may be important to measure. Indeed the complexity illustrated in Figure 1, suggests there may not be a single method that can capture all aspects of child-technology interaction critical to a particular research question. For example, a number of researchers [40,61,117] suggested the combined use methods such as an online survey, time use diary, electronically prompted sampling and onboard logging to answer research questions around attitudes, practices, content and context of use and exposure to short bursts of screen use. Further, Kaye [27] et al. encouraged researchers to consider more than just screen time, and to consider user focused methods. Building on the child-technology interaction model, Figure 2 provides a checklist of considerations to help determine what could be the most suitable method options to investigate children and adolescents’ screen use for a particular study.

Firstly, researchers should consider their specific study aim, the potential study design and what resources are available to them.

Next in considering the complexity of screen use within the child-technology model (Figure 1), researchers should consider the target participants, types of technology, tasks of technology use and interaction aspects of interest, as well as the local context setting and the broader environment of interest, and the time of year/week/day that the technology is likely to be used.

Researchers should also consider what evidence is available for the reliability and validity of the measurement method. Consideration should also be given to the intrusiveness of the method and the impact of this on ecological validity. Similarly, researchers should consider the ease of use of the method, for both participants and researchers and try to minimise the burden, balanced against benefit of the information obtained. 

In considering the ethical considerations some methods may be more or less appropriate for certain kinds of children and families. Importantly, consideration should be given to gaining children’s assent (as well as parent/caregivers’ informed consent), participant and researcher safety (particularly in different settings), and data privacy, which is particularly important for research involving children [18]. 

Finally, researchers should consider if there have been any new advances in measurement method options.

### Scenario Examples Using the Considerations Checklist

To illustrate how the information in this paper can be used by researchers designing studies to investigate the association between screen use and child development the following two research study scenarios illustrate the use of the considerations checklist to determine which measurement method option(s) to use to measure screen use in children and adolescents.

Scenario A: A team of researchers are designing a study with the aim of investigating what types of screen devices young children are using during a typical day and for how long. An observational study design was determined to be the best fit. The team has a budget to purchase equipment as needed and have contacts within a local playgroup centre for participant recruitment. The target participants are children aged 4 years of age. A range of typical technologies could be used by 4-year-olds including TV and tablet devices. The main desired interaction aspects of interest include duration of use and type of screen devices being used. The researchers would like to capture data in multiple locations which could include in the home and outdoors. Based on this scenario measurement options could include a parent reported questionnaire, direct observation or wearable cameras. A parent reported questionnaire could allow for collection of data within a fairly short period of time from a diverse and substantial number of people, however, could be subject to recall inaccuracy and reporting bias leading to overall imprecision. Additionally, it would miss the time the child is in child care. Direct observation could be used for children of this age and could capture any device type; however, it may reduce ecological validity because the presence of the observer may influence the child’s behaviour, and following multiple children for full days would have high researcher burden. A wearable camera could be used for a child of this age and could capture a variety of screen devices, however, there are privacy concerns that should be considered including capturing inappropriate images, capturing third parties, as well as high researcher burden from coding the images. A choice could be made to use wearable cameras, using a camera with a privacy button, giving parents/caregivers the opportunity to review and delete images, and facial blurring software being applied to the images. Advances in machine learning could be used to combat the high researcher burden to code images captured by wearable cameras.

Scenario B: A team of researchers are designing a study with the aim of investigating social connectedness of adolescents during social media use. An observational study design suited the aim. The target participants are adolescents 13–18 years of age. Technology type is the participants’ own smartphones and tablet devices and the social media apps and content they interact with. The tasks focused on include leisure and daily living tasks and the interaction aspects of interest include comments and likes. Adolescents use social media in many different locations and both alone and when with peers. The broader environment considerations include the cultural group and the time considerations include the time of day and proximity to school exams. Based on this scenario, measurement options could include a diary completed by the adolescent or onboard device logging. A diary could be used to ask adolescents about their social media use including platforms used and estimated time of usage but may have social desirability bias and it would be difficult to capture short bursts of exposure. Onboard device logging can measure duration of time on social media by measuring app usage and can capture short bursts of exposure of mobile phone/tablet use, however, the adolescent must own devices with operating software compatible with the measurement app, which may constrain who can participate in the study. Remote digital trace logging at internet service provider or social media platform level could also be used. Digital trace logging allied with machine learning could enable in-depth examination of emotional aspects of interaction. Given the target population, onboard or remote logging, together with machine learning to deal with the large dataset logging can create, could be chosen as the best measurement option for the study aim.

## 6. Conclusions

This paper provides a unique contribution to the field by providing practical support to researchers designing studies to investigate the association between screen use and child health, well-being and development. It provided a conceptual framework for thinking about potentially relevant elements using the child-technology interaction model and outlined some of the challenges faced in capturing contemporary screen use by children and adolescents. It then described the range of available options for measuring screen use by children, providing examples of use, constructs measured and relevant advantages and disadvantages of each method drawn from the literature base and the authors’ own experiences. The paper also provided a checklist and worked example scenarios to support researchers attempting to select the most appropriate method option(s).

Children’s engagement with digital screen technology is complex, as are the aspects of child health, wellbeing and development influenced by interacting with technology. Thus, selecting appropriate measurement method(s) is difficult, but essential to developing better evidence to support guidance on helping children to thrive in a digital world.
